# Mitochondrial Peroxiredoxin III Protects against Non-Alcoholic Fatty Liver Disease Caused by a Methionine-Choline Deficient Diet

**DOI:** 10.3390/antiox12010009

**Published:** 2022-12-21

**Authors:** Jiyoung Park, Nam Hee Kim, Ho Jin Yi, Sue Goo Rhee, Hyun Ae Woo

**Affiliations:** 1 College of Pharmacy, Graduate School of Pharmaceutical Sciences, Ewha Womans University, Seoul 120-750, Republic of Korea; 2 Fluorescence Core Imaging Center, Department of Life Science, Ewha Womans University, Seoul 120-750, Republic of Korea; 3 College of Pharmacy, Graduate School of Applied Science and Technology for Skin Health and Aesthetics, Ewha Womans University, Seoul 120-750, Republic of Korea; 4 The Biochemistry and Biophysics Center, NHLBI, National Institutes of Health, Bethesda, MD 20892, USA

**Keywords:** peroxiredoxin III, reactive oxygen species, non-alcoholic fatty liver disease, methionine-choline deficient diet

## Abstract

Non-alcoholic fatty liver disease (NAFLD) is emerging as the most common chronic liver disease worldwide. In addition, NAFLD may increase the risk of cardiovascular and liver-related diseases, and displays features of metabolic syndrome. In NAFLD, oxidative stress is primarily caused by excessive free fatty acids. The oxidation of fatty acids is usually caused by β-oxidation of mitochondria under normal conditions, resulting in the production of energy. However, when the inflow of fatty acids in NAFLD becomes excessive, the β-oxidation of mitochondria becomes saturated and the oxidation process increases at sites including peroxisomes and microsomes, thereby increasing production of reactive oxygen species (ROS). Thus, hepatic mitochondrial ROS play an important role in the pathogenesis of NAFLD. Eliminating mitochondrial ROS may improve NAFLD, but the underlying mechanism remains unclear. We examined the effect of mitochondrial ROS on NAFLD by focusing on peroxiredoxin (Prx), an antioxidant protein that can remove hydrogen peroxide. The protective effect and pathological phenomenon of mitochondrial peroxiredoxin in methionine-choline deficient diet (MCD)-induced liver injury was assessed in a mouse model of NAFLD. In these mice, mitochondrial peroxiredoxin deficiency significantly increased hepatic steatosis and fibrosis. In addition, ablation of Prx III enhances susceptibility to MCD diet-induced oxidative stress and exacerbates NAFLD progression by promoting inflammation. The binding assay results also showed that Prx III-deficient mice had more severe liver damage than Prx III-abundant mice in MCD diet liver injury models. The present data suggest that mitochondrial peroxiredoxin III could be a therapeutic target for preventing and suppressing diet-induced NAFLD.

## 1. Introduction

Non-alcoholic fatty liver disease (NAFLD) is defined as the presence of excessive fat in the liver without severe alcohol consumption or overt inflammation. The incidence of NAFLD has steadily increased as a result of the dramatic increase in overweight and obese populations over the past few decades, now estimated to be 25.2% globally. NAFLD includes a broad spectrum of liver diseases, such as simple steatosis, NAFL, non-alcoholic steatohepatitis (NASH) cirrhosis and hepatocellular carcinoma (HCC). Unlike cirrhosis or HCC, NAFL and NASH are considered reversible states in which diseases can be alleviated. The exact mechanism by which diseases can be reversed has not been identified [1,2,3]. NASH is reversible to NAFLD and can return to a healthy state. However, after steatohepatitis, it is irreversible, and the risk can be increased. Although NAFLD is the predominant cause of chronic liver disorders, the mechanisms of its development and progression remain incompletely understood. Various scientific groups have attributed these mechanisms to the occurrence of insulin resistance, dyslipidemia, inflammation and apoptosis. NAFLD is one of the most important causes of liver disease worldwide and has emerged as a leading cause of end-stage liver disease in recent decades.

The development of NASH is considered a “two hit” process [4]. The ‘first hit’ leading to liver steatosis results from insulin resistance, which leads to hepatic de novo lipogenesis and impaired fatty acid exportation. The ‘second hit’ consists of the elevated output of reactive oxygen species (ROS) that increases oxidative stress capable of mediating inflammation and cytotoxicity, thereby culminating in NASH and hepatic fibrosis [5]. A ‘third hit’ has also been suggested, based on the fact that overstated oxidative stress produces gradual hepatocyte death and lessens replication of mature hepatocytes and progenitor cell expansion, therefore causing liver cirrhosis and HCC to ensue [6,7]. As a result, fat accumulation in the liver (first hit) increases vulnerability to oxidative stress (second hit), which triggers liver inflammation, endoplasmic reticulum (ER) stress, mitochondrial dysfunction and the inability of hepatocytes to synthesize endogenous antioxidants [8]. Hence, even if NAFLD and NASH are multifactorial diseases, oxidative stress appears to be the major mechanism causing liver injury in NAFLD [9,10]. Excessive production of mitochondrial ROS can damage cell components that include lipids, proteins, and nucleic acids, leading to oxidative stress and ultimately apoptosis [11]. It is widely accepted that the excessive production of mitochondrial ROS is the main cause of the initiation and progression of hepatic steatosis. Thus, the development of NASH which includes this genetic polymorphism is now regarded as a “multiple hit” process [12].

Many causes of cellular stress responses, including oxidative stress and apoptosis, trigger acute phase inflammatory responses and progressive liver damage. Oxidative stress is responsible for the initiation of the immune response to necrosis in a living organism. ROS generated during the oxidative metabolism of fatty acids in mitochondria, peroxisomes, and microsomes are established sources of oxidative stress. Mitochondria are the most important cellular source of ROS, and mitochondrial dysfunction may play a key factor in the pathogenesis of NASH. Although the mechanisms of mitochondrial dysfunction-mediated ROS overproduction results are not clearly understood, emerging data suggest that the second hit causes the promotion of oxidative stress due to ROS overproduction, leading to various insults, such as inflammation and apoptosis, which are typical of NASH and fibrosis. Furthermore, ROS induce the directional migration of resident hepatic pro-fibrogenic cells, resulting in liver fibrosis. We recently showed that mitochondrial peroxiredoxin counteracts oxidative stress in mouse models of fatty liver disease [13,14]. Peroxiredoxins (Prxs) play a substantial role in scavenging cellular peroxides in mammalian cells, with six Prx isoforms (Prx I–VI) found present in the cytosol, mitochondria, and peroxisomes [15]. Prx III is directed toward the mitochondria, as it contains a mitochondrial targeting signal (MTS) at the N-terminal region [16]. It has been reported that Prx III is primarily responsible for reducing the mitochondrial ROS, even in the presence of other enzymes that catalyze the removal of hydrogen peroxide (H_2_O_2_) [17]. Notably, increased ROS production in the liver of Prx III hyperoxidation-induced mice fed with ethanol has been demonstrated; excess ROS was accompanied by the accumulation of inactive Prx III, which aggravates mitochondrial injury [18,19]. These findings indicate that Prx III plays a major role in the development of hepatic steatosis [20]. We previously identified that mitochondrial Prx III protects against alcoholic fatty liver disease in mice by inhibiting oxidative stress and phosphatase and tensin homolog (PTEN) oxidation [21]. Despite these previous findings, much remains unknown regarding the molecular and cellular mechanisms of Prx III in hepatic steatosis.

Here, we review the importance of mitochondrial oxidative stress in the pathogenesis of NASH and the effects of mitochondrial antioxidants on hepatic damage, inflammation, and fibrosis in mice fed a methionine-choline deficient (MCD) diet, a well-defined model of NASH.

## 2. Material and Methods

### 2.1. Animals

Prx III -/- mice were used as previously described [20,21]. Prx III +/+ and -/- mice were generated by breeding heterozygous mice. Ten- to 12-week-old male C57BL/6J mice (Jackson Laboratory, Bar Harbor, ME, USA) were used for the experiments performed according to a protocol approved by the Institutional Animal Care and Use Committee of Ewha Womans University (EWHA IACUC 21-034-t). The mice were housed in a temperature-controlled room (20–22 °C) with alternating 12 h periods of light and dark. C57BL/6J male mice (Jackson Laboratory) were fed an MCD diet for the indicated times. Prx III wild-type and knockout (KO) male mice were fed an MCD diet for the indicated times. They were fed either a normal chow diet (ND) or an MCD diet (L-Amino Acid Diet without choline and methionine, A02082002BR; Research Diets, New Brunswick, NJ, USA) for the indicated periods. Body weight and food intake were measured daily throughout the experimental period. At the end of the feeding trial, the mice were sacrificed, and the liver and blood were collected from each mouse for further analysis.

### 2.2. Measurements of Blood Parameter

Blood was collected from inferior vena cava and plasma was separated by centrifugation at 3000 rpm for 15 min at 4 °C. Plasma alanine aminotransferase (ALT) and aspartate transaminase (AST) concentrations were determined using the EnzyChrom^TM^ assay kits (BioAssay Systems, Hayward, CA, USA) according to the manufacturer’s recommendations.

### 2.3. Immunoblotting

Protein levels in cells and mouse tissues were evaluated using immunoblot analysis. Cells and tissues were lysed with a cold lysis buffer (20 mM HEPES pH 7.0, 0.15 M NaCl, 10% glycerol, 1% Triton X-100, 1 mM EDTA, 1 mM EGTA, 10 mM β-phosphoglycerate, 1 mM Na_3_VO_4_, 5 mM NaF, 1 µg/mL aprotinin, 1 µg/mL leupeptin, and 100 μM phenylmethyl sulfonyl fluoride [PMSF]) using a polytron homogenizer or sonicator. The homogenates were centrifuged at 15,000 rpm, 4 °C for 15 min. After the protein concentration of the lysates from supernatants using the Bradford assay (Bio-Rad, Hercules, CA, USA), lysates were mixed with sample buffer (62.5 mM Tris-HCl pH 6.8, 10% glycerol, 2% sodium dodecyl sulfate, 0.0125% bromophenol blue, 2.5% β-mercaptoethanol) and heated at 95 °C for 5 min. Proteins in samples of sodium dodecyl sulfate (SDS) buffer (3 g/L Tris, 14.35 g/L glycine, 1 g/L SDS) were obtained by SDS-polyacrylamide gel electrophoresis. The proteins were transferred onto an activated 0.45 μm pore size polyvinylidene difluoride (PVDF) membrane (Millipore, Darmstadt, Germany) using methanol with transfer buffer (3.03 g/L Tris, 14.17 g/L glycine, 20% methanol). The membrane was incubated with 5% bovine serum albumin in Tween-20 Tris-buffered saline (TTBS) at room temperature for 20 min using a rocker, followed by incubation at 4 °C overnight on a rocker with antibodies (1:2000 dilution). Immune complexes were detected with horseradish peroxidase (HRP)-conjugated secondary antibodies (Bio-Rad) and enhanced with chemiluminescence reagents (Ab Frontier, Daejeon, Korea) using IQ800 biomolecular imager (GE Healthcare, Uppsala, Sweden). The abundance of target proteins was quantified by densitometric analysis of the immunoblots. Bradford assay data were obtained using a SpectraMax M2 Microplate Reader (Molecular Devices, Sunnyvale, CA, USA) at the Fluorescence Core Imaging Center at Ewha Womans University. Specific antibodies against the Prx isoforms (I–VI), gluthione peroxidase (GPx), catalase, superoxide dismutase (SOD), thioredoxin reductase (TrxR) and sulfiredoxin (Srx) were prepared as previously described [22,23,24]. Membranes were then incubated with primary antibodies against sterol regulatory element-binding protein 1 (SREBP-1), peroxisome proliferator-activated receptor gamma (PPARγ) (both from Santa Cruz Biotechnology, Dallas, TX, USA), transforming growth factor-beta (TGF-β; Abcam, Cambridge, UK), Smad3, phosphorylated Smad3 (p-Smad3), collagen type I alpha 1 chain (COL1A1) and glyceraldehyde-3-phosphate dehydrogenase (GAPDH; all from Cell Signaling Technology, Beverly, MA, USA).

### 2.4. Histology and Immunohistology Assays

Liver tissues fixed in 4% paraformaldehyde were embedded in paraffin using standard procedures. Liver tissue sections were prepared and immunohistochemical staining was performed according to the manufacturer’s instructions. Liver tissue Section 4 µm in thickness were stained with hematoxylin and eosin (H&E) or immunohistochemical characterization was performed using antibody specific to 8-oxo-2′-deoxyguanosine (8-oxo-dG; Trevigen, Inc., Gaithersburg, MD, USA), anti-4HNE (JalCA, Tokyo, Japan), 3-nitrotyrosine (3NT; Millipore, Darmstadt, Germany), and F4/80 (Abcam). Liver sections were stained with Schiff’s reagent (Sigma-Aldrich, St. Louis, MO, USA) for 20 min, followed by counterstaining with hematoxylin solution for 2 min. Sirius Red staining was performed to reveal collagen deposition. The slides were deparaffinized, hydrated with xylene and ethanol, and then incubated with picric acid solution containing 0.1% Fast-green FCF and 0.1% Direct Red 80 for 2 h. All steps were performed at room temperature and the tissues were rinsed with tap water after each step. The stained sections were photographed using a microscope equipped with AxioCam software (Carl Zeiss, Jena, Germany).

### 2.5. Quantitative Real-Time Polymerase Chain Reaction (PCR)

The sample was lysed with 1 mL TRIzol (Invitrogen, Carlsbad, CA, USA) using a homogenizer on ice. Chloroform was added (200 μL) and vigorously vortexed for 15 s, followed by a 3 min rest in order to separate the phenol from lysate, then centrifuged at 12,000× *g*, 4 °C for 15 min. Lysates were separated into three layers. The aqueous top layer that contained RNA was transferred to another tube. Isopropanol was added (500 μL) and the tube was inverted gently four times and kept at −80 °C for 16 h for precipitation of the RNA. Subsequently, the frozen mixture in the tube was melted on ice, then centrifuged at 12,000× *g* and 4 °C for 10 min. The pellet was washed twice with 70% ethanol in RNase-free water, centrifuged at 12,000× *g* and 4 °C for 10 min, and dried. The RNA pellet was resuspended in RNase-free water. RNA purity and concentration were determined using a NanoDrop ND-1000 spectrophotometer (Daemyung, Seoul, Republic of Korea). Total RNA was isolated from cultured cells using TRIzol reagent. Random hexamer primers were added at room temperature and complementary DNA (cDNA) was synthesized using an ABI cDNA synthesis kit (Applied Biosystems, Waltham, MA, USA). The resulting cDNA was subjected to real-time PCR using SYBR Green (Perkin Elmer, Foster City, CA, USA) and an ABI PRISM 7700 system (Perkin Elmer). Glyceraldehyde 3-phosphate dehydrogenase (GAPDH) was used as an internal control. The primer sequences for mouse cDNAs (forward and reverse) are listed in Table 1.

### 2.6. Statistical Analysis

Western blot protein bands were quantified via densitometry using ImageJ 1.501 software (National Institutes of Health, Bethesda, MD, USA). Quantitative analysis of the images for measuring intensity was performed using NIS-Elements software 3.1 (Nikon Instruments Inc., Melville, NY, USA). All values are expressed as the mean ± standard error (S.E). Statistical significance was analyzed via 2-factor ANOVA with Tukey’s test for multiple comparisons using GraphPad Prism software, version 6 (GraphPad, San Diego, CA, USA). Statistical significance was set at *p* < 0.05.

## 3. Results

### 3.1. MCD Diet-Induced Mitochondrial ROS Production Contributes to Accelerated Hepatic Lipid Accumulation

**Figure 1:**
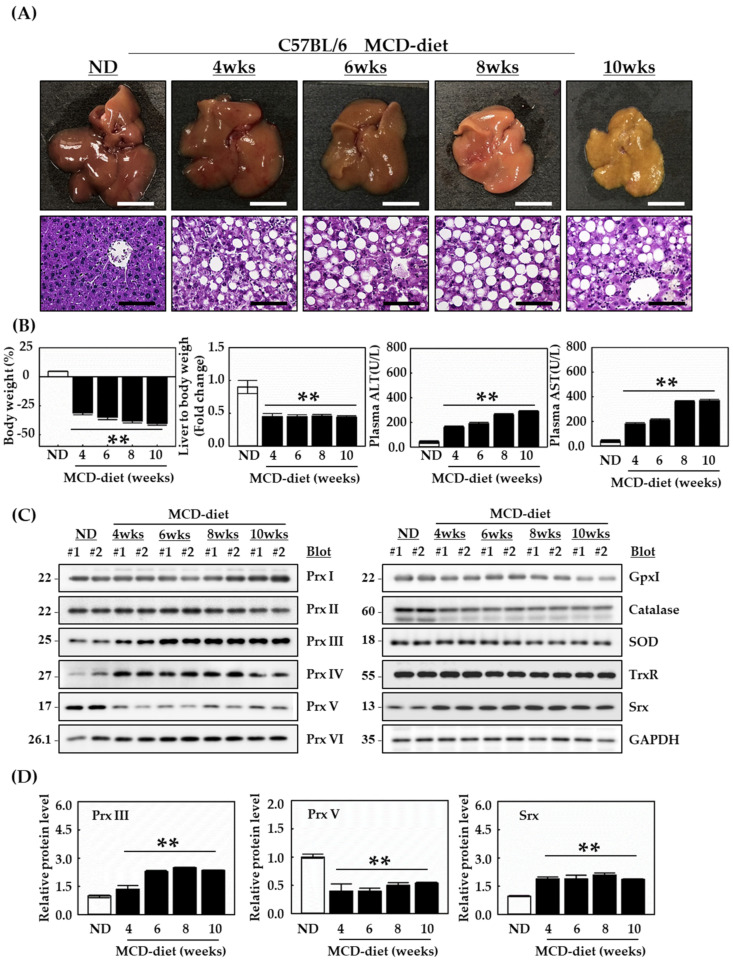
MCD diet-induced hepatic damage and antioxidant protein expression levels in C57BL/6 mice. Male, 8-week-old C57BL/6 mice were fed the ND or MCD diet for 10 weeks. The mice were sacrificed at 4, 6, 8 and 10 weeks. (**A**) H&E staining of representative liver sections at 4, 6, 8 and 10 weeks. Each liver sample was fixed with 4% formaldehyde in phosphate-buffered saline at 4 °C overnight and embedded in paraffin. The liver sections (4 μm thick) were stained using H&E. The scale bar indicates 100 µm, with an original magnification of 400×. (**B**) Mice fed the MCD diet were measured to determine body weight (%) and liver-to-bodyweight ratio. Liver injury was assessed by plasma ALT and AST levels. Proteins from homogenized liver tissue were analyzed by Western blotting. For statistical significance, five liver extracts of the same part in each individual were used for each group. (**C**) Expression levels of Prxs and other antioxidant proteins in the livers of C57BL/6 mice. (**D**) Protein expression levels were normalized against GAPDH protein. Data are means ± SE from three independent experiments, with four to six mice per group ** *p* < 0.01 vs. control group mice fed ND.

NAFLD encompasses a histological spectrum ranging from steatosis to NASH. NAFLD is often associated with increased oxidative stress [25]. To clarify the underlying mechanisms, we used a C57BL/6 mouse model of MCD-induced NAFLD. To investigate whether oxidative liver injury is increased in mice, we first examined liver tissue in these mice. To induce NAFLD, 8-week-old male mice were fed the ND or MCD diet for 10 weeks. The mice were sacrificed at 4, 6, 8 and 10 weeks. Liver and plasma were obtained from each mouse. As expected, the liver size gradually decreased in mice fed the MCD diet for 10 weeks (Figure 1A). Livers from the MCD-fed mice became lighter with time, due to fat accumulation, which indicated the development of NAFLD (Figure 1A). Subsequently, standard H&E staining was used to observe structural changes in the liver. Liver fat content and hepatic lipid droplet number and size increased in the MCD group (Figure 1A). In addition, mice fed the MCD diet gradually developed macrovesicular steatosis more than those fed the ND. At the MCD diet for 10 weeks, neutrophils were concentrated around the portal vein. Mice fed the MCD diet were then measured for body weight (%) and liver-to-body weight (Figure 1B). Mice fed the MCD diet lost body weight instead of being obese. The liver-to-body weight gradually decreased compared with mice fed the ND due to a significantly lower caloric intake. Mice fed the MCD diet were also characterized by a lack of insulin resistance, which is common in patients with NASH [26,27]. Plasma ALT and AST levels, which are indicators of liver injury, were significantly increased in mice fed the MCD diet for 10 weeks, compared with mice fed the ND. To detect the expression levels of Prxs and other antioxidant proteins in the livers of mice fed the MCD, liver extracts were analyzed by Western blotting. Srx, which is an oxidative stress-induced protein downstream of Nrf2, was increased in the MCD diet group compared with the ND diet group. Prx V was reduced and Prx I, II, VI, GPx, SOD, and TrxR did not change in mice fed the MCD diet compared with mice fed the ND diet (Figure 1C,D).

In NAFLD, β-oxidation of mitochondria becomes saturated as fatty acids (FAs) increase. When electrons in this oxidation process are excessively generated and are not smoothly transferred to the mitochondrial respiratory chain, ROS levels are further increased in the mitochondria [28]. These results indicate that Prx III located in mitochondria are important in NAFLD. Therefore, we hypothesized that Prx III might improve obesity-induced fatty liver disease by regulating mitochondrial ROS levels in mouse hepatocytes.

### 3.2. MCD Diet Induces Liver Injury and Oxidative Damage Is Exacerbated by Ablation of Prx III

**Figure 2:**
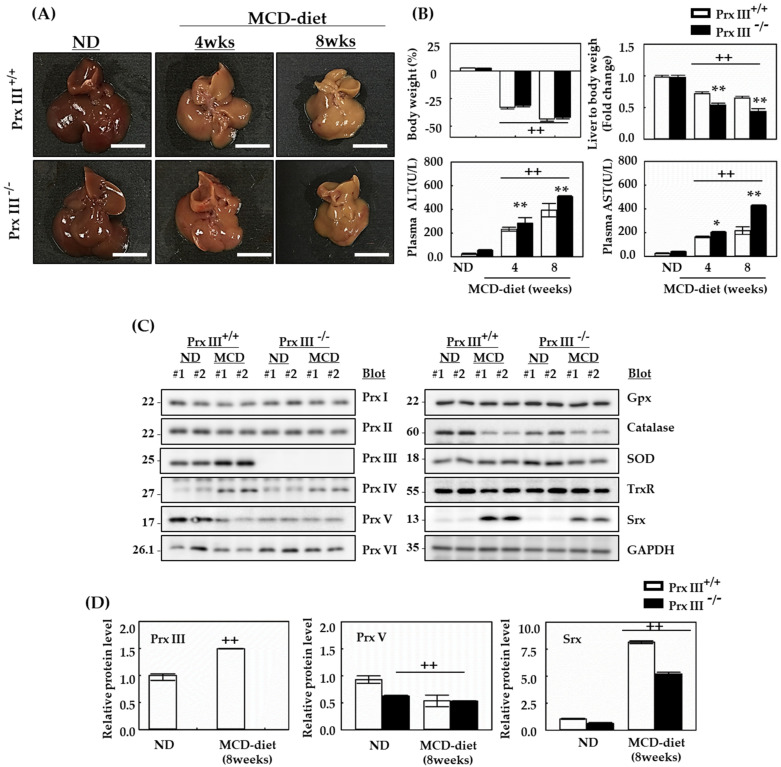
Antioxidant protein expression levels of Prx III +/+ and -/- mice fed the MCD diet. Mice fed the ND or MCD diet were sacrificed after 4 and 8 weeks. (**A**) When the MCD diet was applied for 8 weeks, the size of the liver progressively decreased with time. (**B**) Prx III +/+ and -/- mice fed the MCD diet were examined to determine body weight (%) and liver-to-bodyweight ratio. Liver injury was assessed by determining plasma levels of ALT and AST. Livers of Prx III +/+ and -/- male mice fed the MCD diet were extracted at 8 weeks. Proteins from homogenized liver tissue were analyzed by Western blotting. For statistical significance, five liver extracts of the same part in each individual were used for each group. (**C**) Expression levels of Prxs and other antioxidant proteins in the liver of Prx III +/+ and -/- mice. (**D**) Protein expression levels were normalized against GAPDH protein. Data are means ± SE from three independent experiments, with four to six mice per group. ++ *p* < 0.01 vs. control group mice fed ND, * *p* < 0.05, ** *p* < 0.01 vs. the MCD diet-fed Prx III +/+ mice.

To understand the role of Prx III in the control of NASH-induced dysregulation, we examined the status of various biochemical parameters in MCD-fed and ND-fed mice. Eight-week-old, male Prx +/+ and -/- mice were fed the MCD diet for 4 and 8 weeks. Mice were sacrificed at both times and the liver and plasma were obtained from each mouse. In mice fed the MCD diet, the size of the liver progressively decreased during the 8-week period as the weeks progressed (Figure 2A,B). Prx III +/+, -/- male mice fed the MCD diet lost body weight, and their liver-to-body weight ratio decreased compared with mice fed the ND (Figure 2B), due to a significantly lower caloric intake. Prx III-deficient mice showed no difference in body weight compared with Prx III +/+ mice. However, the liver-to-body weight ratio of Prx III-deficient mice was lower than that of Prx III +/+ mice. Subsequently, liver injury was assessed by measuring plasma levels of liver enzymes (ALT and AST). Both plasma ALT and AST levels were significantly increased in the MCD diet group. In Prx III -/- mice, plasma ALT and AST levels were dramatically higher than those in Prx III +/+ mice fed the MCD diet. To detect the expression levels of Prxs and other antioxidant proteins in the livers of mice fed the MCD diet, liver extracts were subjected to Western blotting. Similar to C57BL/6 mice, Srx and Prx III levels were increased in the MCD diet compared with the ND. Prx V was reduced and Prx I, II, VI, GPx, SOD, and TrxR did not change in the MCD diet compared with the ND diet (Figure 2C,D). Prx III deficiency was confirmed using Prx III antibodies.

As the MCD diet was reproduced in Prx III +/+, -/- mice, several studies have examined the effect of Prx III deficiency on NAFLD.

### 3.3. Deficiency of Mitochondrial Prx III Further Exacerbates Liver Lipogenesis in Mice Fed the MCD Diet

**Figure 3:**
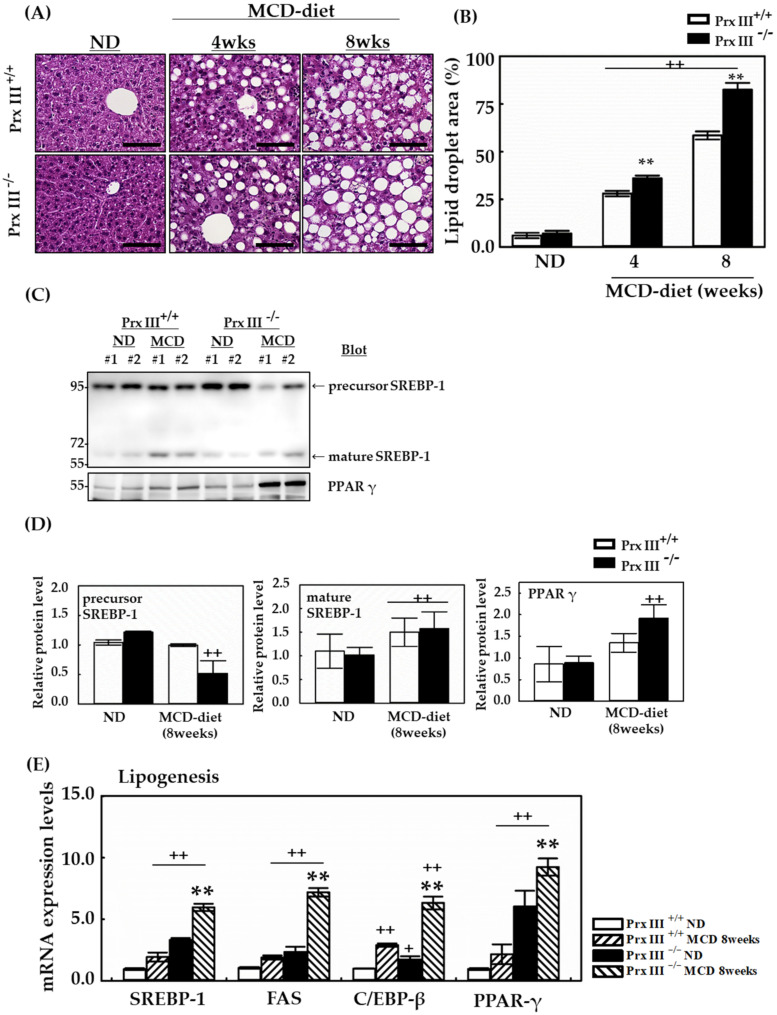
Effects of Prx III deficiency on liver morphology and hepatic lipogenesis in the MCD diet revealed by H&E staining. Prx III +/+ and -/- mice were fed the ND or MCD diet for 4 and 8 weeks. Mice were sacrificed for analyses at both times. The liver was fixed with 4% formaldehyde solution in phosphate-buffered saline at 4 °C overnight and embedded in paraffin. (**A**,**B**) Liver sections (4 μm thick) were stained by H&E and stain deposition was quantified using. NIS-Elements AR 3.1 software. The scale bars indicate 100 µm at original magnifications of 400×. In addition, livers obtained at 8 weeks from mice fed the MCD diet were analyzed by Western blotting. For statistical significance, five liver extracts of the same part in each individual were used for each group. (**C**,**D**) Expression levels of SREBP-1 and PPAR γ in the liver of mice fed the MCD diet. Protein expression levels were normalized against GAPDH protein and are expressed as mean ± SD. Real-time PCR was performed to assess mRNA levels of lipogenesis-related genes in mice fed the MCD diet for 8 weeks. mRNA isolated from liver samples using TRIzol reagent was subjected to RT-PCR and quantitative PCR. Each expression level of mRNA was normalized by GAPDH mRNA. (**E**) mRNA expression levels of lipogenesis-related genes in Prx III +/+ and -/- mice fed the ND or MCD diet for 8 weeks. Data are means ± SE from three independent experiments, with four to six mice per group. + *p* < 0.05, ++ *p* < 0.01 vs. control group Prx III +/+ mice fed ND, ** *p* < 0.01 vs. the MCD diet-fed Prx III +/+ mice.

To examine whether Prx III deficiency affects liver injury in mice fed an MCD diet, a histological examination of liver tissue was performed. The liver fat content and hepatic lipid droplet number and size were significantly increased after 8 weeks of the MCD diet (Figure 3A). In addition, Prx III +/+ and -/- mice fed the MCD displayed diet-induced macrovesicular steatosis compared with ND mice. Lipid accumulation was markedly significant around the central venous region, and the cytoplasm contained numerous microvascular vacuoles in liver sections from Prx III -/- mice fed the MCD diet. Moreover, the level of the mature form of SREBP-1 also increased in liver tissue in mice fed the MCD diet. The content of SREBP-1 was further enhanced by Prx III deficiency, as was the content of FA synthase (FAS), an FA-modifying enzyme that acts downstream of SREBP-1 and PPARγ and which is a critical regulator of adipogenesis. The expression of the C/EBPβ transcriptional activator, which is essential for adipocyte differentiation, was also significantly induced by the MCD diet (Figure 3C–E).

The MCD diet accelerated the maturation of the SREBP1 transcription factor and the expression of lipogenic genes, leading to the development of NAFLD in Prx III-deficient mice.

### 3.4. Ablation of Mitochondrial Prx III Induces Highly Enhanced Oxidative Stress in Mice Fed the MCD Diet

**Figure 4:**
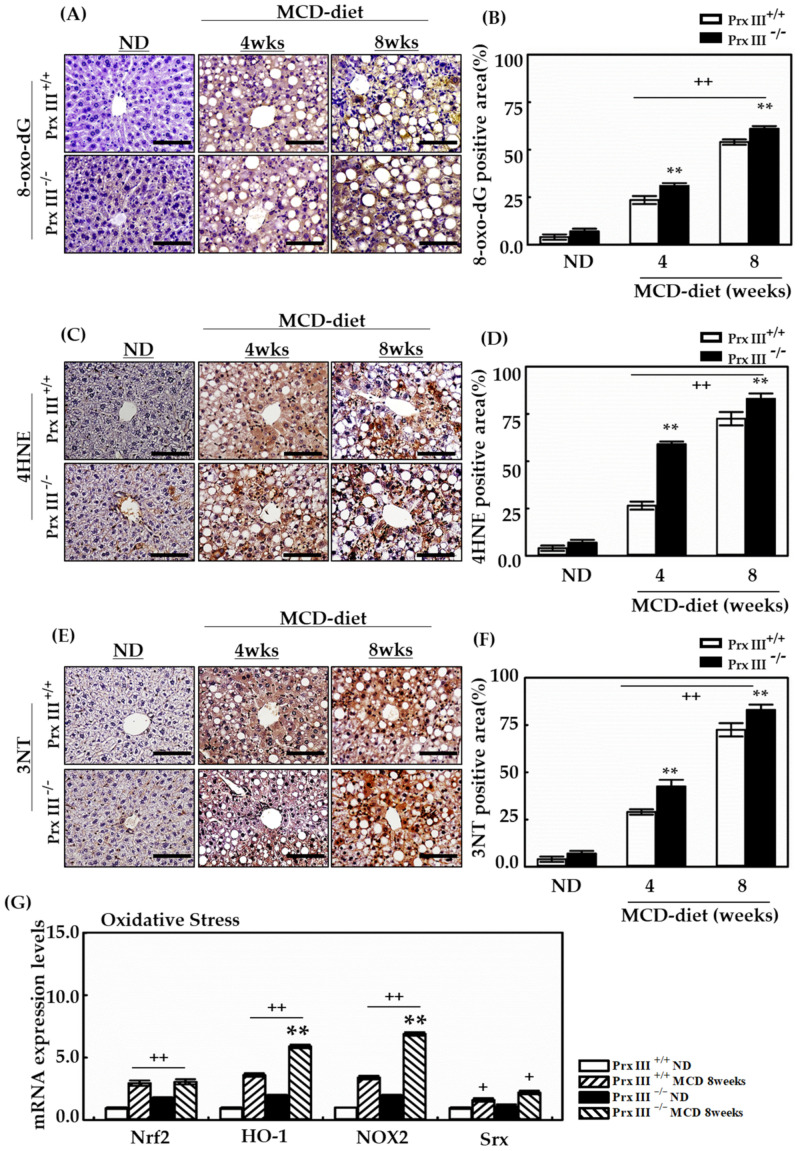
Effects of Prx III on oxidative damage. The effects were assessed in Prx III +/+ and -/- mice fed the MCD diet for 4 and 8 weeks by the staining of 8-oxo-dG, 4HNE, and 3NT, and through determination of mRNA expression levels associated with oxidative stress. Mice were sacrificed at 4 and 8 weeks. Liver tissues were fixed with 4% formaldehyde in phosphate-buffered saline at 4 °C overnight and embedded in paraffin. (**A**–**F**) The 4 μm thick liver sections were stained for 8-oxo-dG, 4HNE, and 3NT as markers of oxidative stress, lipid peroxidation, and tyrosine oxidation marker, respectively. NIS-Elements AR 3.1 software was used to quantify staining. The scale bars indicated 100 µm at original magnifications o 400×. Real-time PCR assessed mRNA levels of oxidative stress-related genes were measured in mice fed the MCD diet for 8 weeks. mRNA from liver tissues, using TRIzol reagent, was subjected to RT-PCR and quantitative PCR. Each expression level of mRNA was normalized by GAPDH mRNA. (**G**) The mRNA expression levels of oxidative stress-related genes in Prx III +/+ and -/- mice fed ND or the MCD diet for 8 weeks. Data are means ± SE from three independent experiments, with four to six mice per group. + *p* < 0.05, ++ *p* < 0.01 vs. control group mice fed the ND, ** *p* < 0.01 vs. Prx III +/+ mice fed the MCD diet.

Oxidative stress is implicated in the pathogenesis of NAFLD and can be the cause of inflammation. Given the finding that the MCD diet induced oxidative stress in the liver, which resulted in an increased accumulation of protein carbonylation [29], we determined the effects of Prx III on MCD diet-induced liver oxidative stress. Liver sections prepared from mice fed the MCD or ND diet were examined for 8-oxo, 4-hydroxynonenal (4-HNE), and 3-NT. The hallmark oxidative DNA damage was clearly elevated in the central vein area, as determined by 8-oxo-dG staining in MCD-fed Prx III -/- mice (Figure 4A,B). These mice also displayed the production of the 4-HNE periportal lipid peroxidation product due to oxidative stress (Figure 4C,D). The formation of 3-nitrotyrosine protein adducts is an indication of peroxynitrite formation. High levels of 3-NT staining were observed in the livers of mice fed the MCD diet, and more intense staining of oxidative stress markers was observed in the livers of MCD-fed Prx III -/- mice (Figure 4E,F). The levels of some antioxidant enzymes were also assayed by real-time PCR. The MCD diet increased the mRNA levels of Nrf2, HO-1, NQO1, and Srx in each Prx III -/- group(Figure 4G). MCD diet feeding in mice increased levels of HO-1 or NOX2 two-fold in Prx III -/- livers compared with Prx III +/+ livers, and induced similar changes in hepatic Srx mRNA. These findings are consistent with those of a previous report (19) and with the increased mRNA expression levels of nuclear factor-erythroid factor 2-related factor 2 (Nrf2), heme oxidase-1 (HO-1), NADP oxidase 2 (NOX2) and sulfiredoxin (Srx). The findings suggest that Prx III deficiency exacerbates oxidative stress, which may mediate liver injury.

### 3.5. Ablation of Mitochondrial Prx III Promotes MCD Diet-Induced Inflammatory Responses and Fibrosis in Mice Liver Tissues

**Figure 5:**
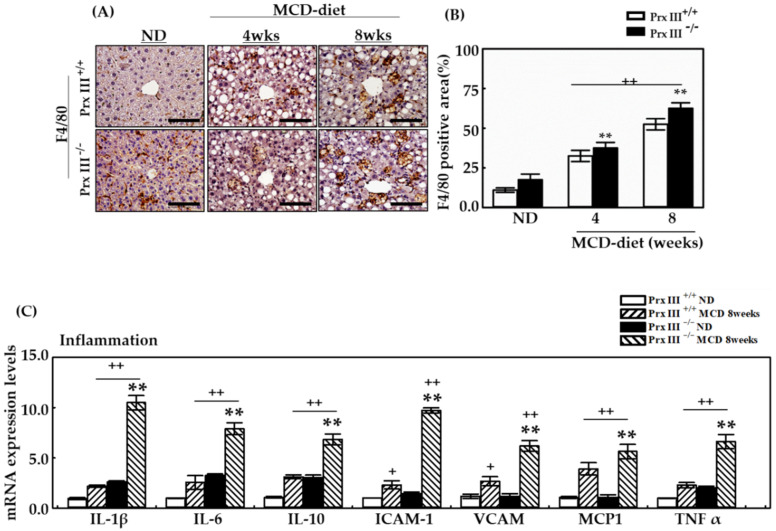
Effects of Prx III deficiency on hepatic inflammation through F4/80 staining and mRNA expression levels associated with liver inflammation in MCD diet. Prx III +/+ and -/- mice were fed the ND or MCD diet for 4 and 8 weeks. Mice were sacrificed at 4 and 8 weeks. The liver from each mouse was fixed with 4% formaldehyde in phosphate-buffered saline at 4 °C overnight and embedded in paraffin. The liver sections (4 μm thick) were stained for F4/80 as a major macrophage infiltration marker. (**A**,**B**) F4/80 staining in liver tissues from Prx III +/+ and Prx -/- mice fed the ND or MCD diet for 4 and 8 weeks was quantified by NIS-Elements AR 3.1 software. The scale bars indicate 100 µm at original magnifications of 400×. Real-time PCR was performed to assess mRNA levels of liver inflammation-related genes in the ND and MCD diet groups at 8 weeks. mRNA from the liver using TRIzol reagent was subjected to RT-PCR and quantitative PCR. Expression levels of mRNA were normalized to that of GAPDH mRNA. (**C**) The mRNA expression levels of liver inflammation-related genes in Prx III +/+ and -/- mice fed the ND or MCD diet for 8 weeks. Data are means ± SE from three independent experiments, with four to six mice per group. + *p* < 0.05, ++ *p* < 0.01 vs. control group mice fed ND, ** *p* < 0.01 vs. the MCD diet-fed Prx III +/+ mice.

NAFLD is characterized by the absence of or mild chronic portal inflammation [30]. In addition, histological liver inflammation plays an important role in NASH development [31]. The activation of circulating monocytes and accumulation of macrophages in the liver are important pathophysiological features in patients with NAFLD. Macrophages play an important role in the initial innate immune response to infection. The two main macrophage categories are infiltrating and tissue-resident macrophages. Infiltrating macrophages are derived from monocytes that circulate throughout the body and are recruited to tissues when an inflammatory reaction occurs. In contrast, tissue-resident macrophages are always localized within one tissue and defend against infection or injury. To investigate the effect of Prx III deficiency on hepatic inflammation in NAFLD, immunohistochemical staining of liver sections of Prx III +/+ mice was performed. F4/80 staining in liver tissue reveals macrophage infiltration expressed on the membranes of macrophages in response to the MCD diet. Macrophage infiltration was significantly increased in liver tissue of 8-week-old male Prx III +/+ mice fed the MCD diet for 4 and 8 weeks compared with ND mice. Prx III-deficient mice displayed greater recruitment of macrophages than Prx III +/+ mice at 4 and 8 weeks. In particular, macrophage recruitment was further increased in Prx III-deficient mice compared with Prx III +/+ mice fed the MCD diet for 8 weeks (Figure 5A,B).

To investigate the inflammation in the liver induced by the MCD diet, we measured the mRNA expression of several inflammation-related cytokines in MCD-fed and ND mice. Quantitative RT-PCR analysis revealed that the MCD diet induced a dramatic increase in pro-inflammatory cytokines (IL-1β, IL-6, and TNFα), anti-inflammatory cytokines (IL-10), and cell adhesion molecules intercellular adhesion molecule 1 (ICAM-1) and vascular cell adhesion protein (VCAM) in Prx III -/- mouse livers compared with Prx III +/+ mice. In addition, monocyte chemoattractant protein-1(MCP1) was induced in the livers of Prx III-/-mice (Figure 5C). Our data suggest that Prx III plays a critical role in MCD diet-induced liver injury inflammation, and may play a role in the attenuation of hepatic fibrosis.

**Figure 6:**
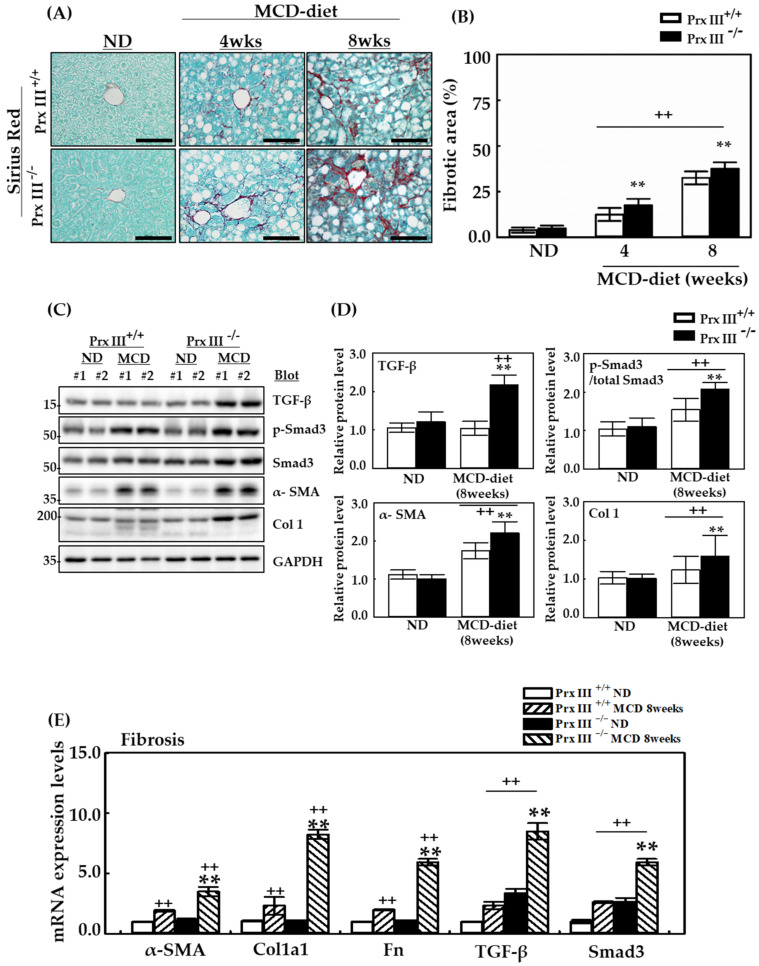
Effects of Prx III deficiency on hepatic fibrosis using Sirius Red staining and mRNA expression levels associated with liver inflammation. Prx III +/+ and -/- mice were fed the ND or MCD diet for 4 and 8 weeks. Liver tissues obtained from mice sacrificed at 4 and 8 weeks were fixed with 4% formaldehyde in phosphate-buffered saline at 4 °C overnight and embedded in paraffin. The liver sections (4 μm thick) were stained with Sirius Red to reveal the major collagen deposition marker. (**A**,**B**) F4/80 staining in liver tissues from Prx III +/+ and Prx -/- mice fed the ND or MCD diet for 4 and 8 weeks were quantified by NIS-Elements AR 3.1 software. The scale bars indicate 100 µm, with original magnifications of 400×; Expression levels of fibrosis marker proteins in the liver of Prx III +/+ and -/- mice. (**C**) Protein expression levels normalized against GAPDH protein. (**D**) Real-time PCR was performed to assess mRNA levels of liver inflammation-related genes in mice fed the ND or MCD diet for 8 weeks. mRNA isolated from liver tissues using TRIzol reagent was subjected to RT-PCR and quantitative PCR. Each expression level of mRNA was normalized to GAPDH mRNA. (**E**) The mRNA expression levels of liver fibrosis-related genes in Prx III +/+ and -/- mice fed the ND or MCD diet for 8 weeks Data are means ± SE from three independent experiments, with four to six mice per group. ++ *p* < 0.01 vs. control group mice fed ND, ** *p* < 0.01 vs. the MCD diet-fed Prx III +/+mice.

Liver fibrosis is a major manifestation of advanced NASH and represents the greatest risk factor for HCC pathogenesis [32]. To evaluate the role of mitochondrial peroxiredoxin III in fibrosis during NASH development, Prx III +/+ and -/- mice were fed the ND or MCD diet for eight weeks. Sirius Red staining revealed that the fibrotic area was significantly more extensive in Prx III -/- mice than in +/+ mice. Staining was conspicuously localized around the portal vein and throughout the lobules in a pericellular distribution in tissues from mice fed the MCD diet (Figure 6A,B). The levels of transforming growth factor-beta (TGF-β), a potent fibrogenic cytokine, and Col 1, a representative TGF-β-targeting collagen gene, were significantly higher in Prx III -/- mice fed the MCD diet than in Prx III +/+ mice fed this diet (Figure 6C).

These results, along with mRNA expression levels of alpha smooth muscle actin (α-SMA), collagen (Col), Fn1, TGF-β and Smad3 suggest that Prx III deficiency induces hepatic fibrosis, which may mediate liver injury (Figure 6D). Thus, important therapeutic targets, such as mitochondrial peroxiredoxin III, that reduce inflammatory cytokines and mitochondrial β-oxidation in the liver may prevent progression from steatosis to steatohepatitis. The findings indicate a potentially rational therapy for the treatment of NAFLD.

## 4. Discussion

NAFLD features the accumulation of fat in the liver in the absence of alcohol consumption. NAFLD is becoming the most common cause of liver disease worldwide [33]. NAFLD correlates strongly with obesity, type II diabetes and other components of metabolic syndrome [34,35,36]. Although the exact mechanisms underlying the development of NAFLD remain unknown [37], extensive lipid accumulation and lipid peroxidation-induced oxidative stress are considered major factors, causing cytotoxicity and exacerbated hepatopathy [38]. Thus, oxidative stress has been implicated in the pathogenesis of NAFLD and NASH, and the involvement of ROS has been suggested. Disequilibrium in lipid metabolism induces oxidative stress via the overproduction of ROS, which affects ROS generation in the mitochondria, ER and NOX. In NAFLD, mitochondria have been extensively studied concerning lipid metabolism and are one of the most important sources of ROS. However, the function of the hepatic antioxidative system in lipid metabolism has not yet been characterized. To examine the MCD diet-induced changes in hepatic lipid metabolism in mice deficient in Prx III, we quantified the mRNA expression of several key transcription factors and their prototypical target genes involved in lipid metabolism in the liver.

The present findings reveal the crucial role and histopathological features of Prx III as a regulator of hepatic lipid metabolism that thereby mitigates the development of hepatic steatosis. A lipogenic MCD diet promotes intrahepatic lipid accumulation in rodents by increasing hepatocyte FA uptake and decreasing very-low-density lipoprotein-triglyceride secretion [39]. In the present study, Prx III-deficient mice were fed an MCD diet for 4 to 8 weeks. Hepatosteatosis was more severe in Prx III-deficient mice and much less severe in Prx III +/+ mice after being fed the MCD diet for 8 weeks (Figure 2). Furthermore, increased AST and ALT levels were observed in Prx III-deficient mice fed the MCD diet (Figure 3), indicating increased hepatocellular injury in these mice. These phenotypic changes support the hypothesis that Prx III mediates NAFLD development. Oxidative stress plays a key role in the development and progression of NAFLD [40] and fibrosis is often observed in diet-induced NAFLD [41]. Markers of oxidative stress, such as 8-oxo-dG, 4-HNE, and 3-NT, were measured in the liver tissues. Immunohistochemical analyses showed that the expression of oxidative stress adducts was further increased by the MCD diet in Prx III -/- mice (Figure 5). These results indicate that ablation of Prx III exacerbates oxidative stress. Steatosis and fibrosis are two pathological processes associated with NAFLD [42]. H&E and Sirius Red staining, capable of evaluating NAFLD activity based on steatosis, lobular inflammation and hepatocellular ballooning, were performed to evaluate hepatic histopathology and fibrosis. Considerably more lipid accumulation was observed in Prx III -/- mice than in Prx III +/+ mice. MCD diet-inducted micrometers clearly showed aortic steatosis, lobular inflammation and hepatocellular ballooning and worsened in PrxIII-deficient mice. In addition, Sirius Red quantification demonstrated that collagen was mainly accumulated in the livers of mice fed the MCD-induced diet; deposition was accelerated in Prx III-deficient mice. Further analysis of the liver lysates showed increases in the lipid metabolism marker SREBPs (Figure 3C) and the fibrogenesis markers TGF-β1 and collagen type I (COL1) in Prx III-deficient mice. Similarly, PPARγ, which regulates FA oxidation, was significantly upregulated. The mRNA levels of C/EBP-β, PPARα, TGF-β1, and COL1 showed the same trend as the protein levels (Figure 3 and Figure 6). These results indicate that mitochondrial peroxiredoxin III effectively improves lipid metabolism and inhibits fibrogenesis, and that Prx III deficiency increases the susceptibility of mice to MCD diet-induced hepatosteatosis and liver injury.

## 5. Conclusions

This study investigated the protective effects of mitochondrial Prx III on MCD diet-induced hepatotoxicity in Prx III-deficient mice. The goal was to find a possible therapeutic application to degenerative diseases, as well as to provide new strategies for the prevention and treatment of NAFLD. Our data implicate mitochondrial Prx III as a therapeutic target for the prevention and treatment of fatty liver diseases. It is also noteworthy that mitochondrial Prx III may offer therapeutic potential for ameliorating the severity of NAFLD and hepatic dysfunction by suppressing oxidative damage.

## Figures and Tables

**Table 1:** Primer sequences of target genes for real-time reverse transcription polymerase chain reaction (RT-PCR).

Genes	Forward Primer	Reverse Primer	Accession No.
SREBP	CATCGACTACATCCGCTTCTTACA	GTCTTTCAGTGATTTGCTTTTGTGA	NM_001358315.1
FAS	TGGTGGGTTTGGTGAATTGTC	GCTTGTCCTGCTCTAACTGGAAGT	NM_007988.3
C/EBP-β	GCCGAGATAAAGCCAAACAA	CCTTGACCAAGGAGCTCTCA	NM_007679.4
PPAR-γ	TTTTCAAGGGTGCCAGTTTC	TTATTCATCAGGGAGGCCAG	NM_001308354.1
Nrf 2	CTCTCTGAACTCCTGGACGG	GGGTCTCCGTAAATGGAAG	NM_010902.5
HO-1	CACGCATATACCCGCTACCT	CCAGAGTGTTCATTCGAGCA	NM_010442.2
NOX2	ACTCCTTGGGTCAGCACTGG	GTTCCTGTCCAGTTGTCTTCG	NM_007807.5
Srx	GGAAGGAAGAAAGGAGATGG	AGAGTTCAGGCTATGGGGAT	NM_029688.5
IL-1β	TCGTGCTGTCGGACCCATAT	GTCGTTGCTTGGTTCTCCTTGT	NM_008361.4
IL-6	ACAACCACGGCCTTCCCTACTT	CACGATTTCCCAGAGAACATGTG	NM_031168.1
IL-10	GCATGGCCCAGAAATCAAGG	AGGGGAGAAATCGATGACAGC	NM_010548.2
ICAM-1	GCCTTGGTAGAGGTGACTGAG	GACCGGAGCTGAAAAGTTGTA	NM_010493.3
VCAM	TGCCGAGCTAAATTACACATTG	CCTTCTTGGAGGGATGTACAGA	NM_011693.3
MCP1	GCTCAGCCAGATGCAGTTAA	TCTTGAGCTTGGTGACAAAAACT	NM_011333.3
TNFα	GCCACCACGCTCTTCTG	GGTGTGGGTGAGGAGCA	NM_013693.3
α-SMA	TCGCTGTCAGGAACCCTGAGACG	ATCATCACCAGCGAAGCCGGC	NM_007392.3
Col1a1	ACCTGTGTGTTCCCTACTCA	GACTGTTGCCTTCGCCTCTG	NM_007742.4
Fn1	CAACAACCGGAATTACACCG	GGTCTCGGAGCTGGGAGTAG	NM_001276413.1
TGFβ	CCGCAACAACGCCATCTATG	CTCTGCACGGGACAGCAAT	NM_009368.3
Smad3	GTCAAAGAACACCGATTCCA	TCAAGCCACCAGAACAGAAG	NM_016769.4
GAPDH	AGAACATCATCCCTGCATCC	GGTCCTCAGTGTAGCCCAAG	NM_001289726.1

## Data Availability

The data is contained within the manuscript.
